# Traditional, Indigenous Apple Varieties, a Fruit with Potential for Beneficial Effects: Their Quality Traits and Bioactive Polyphenol Contents

**DOI:** 10.3390/foods9010052

**Published:** 2020-01-05

**Authors:** Lidija Jakobek, Jozo Ištuk, Ivana Buljeta, Sandra Voća, Jana Šic Žlabur, Martina Skendrović Babojelić

**Affiliations:** 1 Department of Applied Chemistry and Ecology, Faculty of Food Technology Osijek, Josip Juraj Strossmayer University of Osijek, HR-31000 Osijek, Croatiaivana.buljeta@ptfos.hr (I.B.); 2 Faculty of Agriculture, University of Zagreb, HR 10000 Zagreb, Croatia; svoca@agr.hr (S.V.); jszlabur@agr.hr (J.Š.Ž.); mskendrovic@agr.hr (M.S.B.)

**Keywords:** internal quality traits, external quality traits, HPLC, flavonols, dihydrochalcones, phenolic acids, flavan-3-ols, anthocyanins

## Abstract

Earlier studies suggested that traditional apple varieties have quality traits well accepted by consumers and beneficial effects on human health. The aim was to collect 25 traditional apple varieties grown in Croatia and to determine, for the first time in so many details, their external (weight, height, width, shape, color), internal quality traits (firmness, starch decomposition index, maturity index, soluble solid concentration, total acids, soluble solid/total acids ratio, pH), and seed characteristics. In addition, individual polyphenols were determined in the flesh and peel, by using RP-HPLC. All was compared to the commercial variety ‘Idared’. Quality parameters of these varieties were similar to those of the commercial variety. The flesh and peel contained flavan-3-ols, dihydrochalcones, phenolic acids, and flavonols, while anthocyanins were additionally found in the peel. Total polyphenols in the peel (536–3801 mg kg^−1^ fresh weight (FW)) and in the flesh (79–1294 mg kg^−1^ FW) of the majority of varieties were higher than in the commercial variety. Principal component analysis showed possible clustering according to polyphenol amounts. According to the observed diversity of quality traits and bioactive polyphenol contents, the traditional varieties have potential for consumer acceptance and increased cultivation.

## 1. Introduction

Apples are among the most consumed fruits in the world. After China, the European Union is one of the biggest producers of apples. Inside the EU, the biggest producers are Poland, Italy, France, and Germany [1]. The total apple production of EU was 13.8 million tons in 2018 [2]. The dominate commercial varieties in the EU are ‘Golden Delicious’, ‘Gala’, and ‘Idared’, although many other varieties are grown too [3]. Apples are profitable fruit but prices can vary according to the year and country [3]. Besides being produced and consumed in large amounts, apples are important due to their nutritional value. Namely, apples are rich in different nutrients and have a significant amount of bioactive compounds such as polyphenols [4,5,6,7,8,9]. Since they are often consumed, apples are also an important source of bioactive polyphenols in the human diet. Because of this importance, apples and their beneficial effects are being intensively studied [10,11,12].

Over the centuries, numerous different varieties of apples with different quality traits have been developed [13]. Quality traits important to consumer acceptance include certain internal and external quality indicators [13]. External indicators of quality are the height, width, shape, and weight of the fruit. Internal quality indicators may be fruit firmness, a maturity index, a starch degradation index, soluble solid concentration, acid content, and the ratio of the soluble solid concentration to the total acid concentration [13]. These parameters not only include indicators of the state of maturity of the fruit (via the maturity index, the fruit firmness, or the starch index), but also include traits that may relate to consumer acceptance and taste preferences, such as the soluble solids / total acid ratio which is related to the sweetness of the fruit. Another set of indicators of quality that has become very important is the amount of various polyphenolic compounds [4]. As a quality trait, polyphenols have become important due to their positive bioactivities [14,15,16]. Polyphenols in apples belong to different subgroups. These are flavan-3-ols, dihydrochalcones, phenolic acids, flavonols, and anthocyanins [5,17].

The quality traits of commercial apples grown in large quantities such as ‘Fuji’, ‘Golden Delicious’, ‘Gala’, and ‘Idared’ are well known [4,18,19] and according to consumer acceptance and demands, firmness, crunchiness, and sweetness are highlighted as important quality traits of apples [20,21,22]. But in the past, many different apple cultivars have been developed that are difficult to find in today’s consumer world [23,24,25]. These old, traditional, indigenous varieties are not well known, have been neglected, and are grown only in small quantities, mostly in small orchards. Some of those old apple varieties are preserved in germplasm banks of some countries or regions [23] but there are some that have not yet been registered. Since there is growing consumer awareness for locally grown apples [26], these apples could become important in the future.

There is much that remains unknown about these old, traditional, indigenous varieties. Quality indicators are not as well known for these varieties as they are for the commercial varieties [4,18,19]. Regarding polyphenols, it is known that old apple varieties have the same groups of polyphenols as commercial varieties [7,24,25]. But the amounts of polyphenols are not known as well as they are for commercial apple varieties. Old varieties have shown that they contain more polyphenols than commercial varieties [6] or in some studies, the amounts in old and new varieties were similar [5]. In the study of Feliciano et al. [27], both traditional and exotic apple varieties from Portugal showed high amounts of polyphenols. Although old varieties showed higher polyphenol content, it should be noted that environmental conditions can influence polyphenol amounts. Namely, it was shown for black currants that growing in shade caused lower polyphenol content in comparison to growing in normal conditions [28]. Lower crop load on apple trees caused higher polyphenol content [29]. The geographical locations of orchard can influence the color of apples too [30]. In addition, differences between conventional and organic growing might also cause some differences in polyphenol content. Apples grown organically or conventionally showed different polyphenol profile but the polyphenol content was similar [31]. Thus, it is recognized that agricultural practice could impact polyphenols in apples.

The importance of old varieties of apples can be seen from studies that highlight their beneficial effects when compared to those of commercial ones. Namely, old apple varieties have been mentioned as varieties better tolerated than new varieties by individuals who have developed intolerance to apples [32]. Furthermore, a positive feature of these old varieties is a higher amount of polyphenols, although those conclusions need to be studied further in vivo [32]. Another study has also shown that less allergenic genotypes of apples are old varieties developed before the so-called “green revolution”, a period in which genetic improvement of plants was accelerated [33]. All those facts confirm the importance of preserving old, traditional apple varieties. Accordingly, it should be mentioned that there is a growing awareness of the nutritional values of these old varieties as well as their potential for different quality traits such as a diversity of colors, shape, or tastes. That is why there is a growing interest in old, indigenous varieties of apples [23,34] as well as many other fruits or cereals [35,36,37].

The aim of this study was to collect traditional, indigenous apple varieties grown in orchards in Croatia and determine their internal and external quality parameters (including sixteen quality treats and color). To the best of our knowledge, the quality traits of these apples were not previously studied. The other aim was to determine the amounts of the various polyphenolic compounds found in the peel and flesh of the apples. For some of the varieties studied here, polyphenols have never been studied before. All of the quality parameters and polyphenols were compared with the commercial cultivar ‘Idared’.

## 2. Materials and Methods

### 2.1. Chemicals

Polyphenol standards were purchased from several firms. From Sigma-Aldrich (St. Louis, MO, USA), the standards obtained were (+)-catechin hydrate (C1251), (-)-epicatechin (E1753), quercetin dihydrate (Q0125), quercetin-3-rutinoside hydrate (R5143), quercetin-3-glucoside (17793), *p*-coumaric acid (C9008), and chlorogenic acid (C3878), and from Extrasynthese (Genay, France) procyanidin B1 (0983), procyanidin B2 (0984), quercetin-3-*O*-galactoside (1027 S), quercetin-3-*O*-rhamnoside (1236 S), phloretin-2’-*O*-glucoside (1361 S), phloretin (1360 S), cyanidin-3-galactoside chloride (O923 S). Orto-phosphoric acid (85% HPLC-grade) was from Fluka (Buchs, Switzerland). Methanol (HPLC grade) was from J.T. Baker (Gliwice, Poland).

### 2.2. Apple Samples

Around two kg of each variety of apple were harvested from orchards in Croatia (an orchard in the town Donji Mihaljevci (45°22’47.6”N 17°40’34.3”E) owned by Mirko Veić, and orchards in the town Gornji Tkalec (45°58’24.0”N 16°27’12.0”E) and the village Rude (45°46’35.6”N 15°40’35.8”E)) in the following varieties: ‘Crvenka’, ‘Crveni Boskop’, ‘Pisanika’, ‘Lještarka’, ‘Božićnica’, ‘Božićnica 2’, ‘Cox’s Orange Pippin’, ‘Ivanlija’, ‘Boskop’, ‘Bobovac’, ‘Slavonska srčika’, ‘Kolerova srčika’, ‘Batulenka’, ‘Gravenstein’, ‘Kandil Sinap’, ‘Citronka’, ‘Zimnjara’, ‘Zlatica’, ‘Mašanka’, ‘Kanada’, ‘Gloria Mundi’, ‘Zelenika’, ‘Krastavka’, ‘Adamova zvijezda’, and ‘Wild apple’. The variety Božićnica was harvested from two orchards (Donji Mihaljevci and Gornji Tkalec), so the names used here are ‘Božićnica’ and ‘Božićnica 2’, respectively. The commercial variety ‘Idared’ was harvested in the orchard in the village of Rude (Samobor). This variety was chosen because it is one of the most often grown and popular commercial varieties in Croatia and in this study it served as a control. Apples of each variety were divided into two groups. The group of apples for quality determination consisted of approximately one kg of each variety. It was transferred to the laboratory of the Faculty of Agriculture, University of Zagreb, and kept in a refrigerator before internal and external quality traits were determined. The other group of approximately one kg of each variety was transferred to the laboratories of the Faculty of Food Technology, University of Osijek, where they were kept in a refrigerator for no more than 5 days before extracts for the determination of polyphenolic compounds were made. Polyphenol contents of the varieties ‘Bobovac’, ‘Batulenka’, ‘Mašanka’, and ‘Krastavka’ were never studied before to the best of our knowledge. The quality traits and polyphenolic compounds of all varieties were never studied before in so much detail.

### 2.3. External Quality Determination

All apples (5 to 10 apples) in the quality determination group were measured for weight, height, and width. The weight of the fruit was determined by a digital two-decimal laboratory scale (OHAUS Corporation, Parsippany, NJ, USA) and expressed in grams (g). The height and the width of the fruit were measured by the digital caliper (Somet, Czech Republic), and the values were expressed in millimeters (mm). From the height and width measurements, the fruit shape index was represented by the ratio of height/width. Means were calculated for each variety. Color was measured according to CIE Lab system on a colorimeter (PCE-CSM2, PCE Instruments UK Ltd, Hamble-le-Rice, UK). Colorimeter calibration was done with black and white plates supplied with the instrument.

### 2.4. Internal Quality Determination

All apples (5 to 10 apples) in the quality determination group were examined to determine their firmness by using a penetrometer (PCE-PT200, PCE Instruments, Southampton, UK) with 11 mm probe as the average value from four measurements made at opposite fruit sides at the equatorial fruit zone. The values were expressed in kg∙cm^−2^. These apples were then cut in half and the starch index was determined by using a starch-iodine test. Apples were then peeled, cut, and cored, and the seeds were removed from cores. The seeds were used for seed characteristic determination and the remaining apple flesh was mixed and squeezed to get juice. The juice was used for internal quality determination. A refractometer (ATAGO PAL-1, Atago, Tokyo, Japan) was used to measure the soluble solid concentration in apple juice and expressed as °Brix. Furthermore, total acids were measured with titration by neutralization with 0.1% NaOH. Data were expressed as % of malic acid. Additionally, the pH was measured with pH meter (Testo 205, AG, Testo, Lenzkirch, Germany) in the apple juice. All results were expressed as means ± standard deviations.

### 2.5. Seed Characteristics Determination

All seeds were inspected to determine which were healthy and which were bad seed, and the number of each were counted. The resulting total seed number per apple, total healthy seed number per apple, and bad seed number per apple were expressed as mean ± standard deviation. For each apple, each seed as well as all seeds were weighed by the four-decimal analytical scales (KERN® Analytical balance AES-C/AEJ-CM, Kern, Balingen, Germany). The total seed weight and one seed weight were expressed as means ± SD.

### 2.6. Polyphenol Extraction

For the apples in the polyphenol determination group (about one kg of each variety), the procedure was as follows. The apples were peeled with a commercial peeler. For each variety, the peel was pooled together and homogenized with the help of a coffee grinder. The remaining apple flesh was then cut into four equal parts, the seeds were removed and the flesh pieces were pooled together. The flesh was homogenized with a stick blender. These samples were frozen, stored at −18 ° C and extracts were made the second day.

Briefly, for each extract, 0.2 g of peel or flesh was weighed and added to a plastic cuvette. Then, 1.5 mL of solvent consisting of 80% methanol and 20% water was added into each cuvette. The extraction was performed on an ultrasonic bath (Bandelin Sonorex RK 100, Berlin, Germany) for 15 min. The solution was centrifuged (Eppendorf Minispin centrifuge, Eppendorf, Hamburg, Germany) for 10 min at 10,000 rpm. Extracts were separated from the residue. Two parallel samples were prepared for each variety. Extracts were filtered through a 0.45 μm PTFE filters before injection into the HPLC system.

### 2.7. Reversed-Phase High-Performance Liquid Chromatography with Photo-Diode Array Detection (HPLC-PDA)

For the characterization of individual polyphenols, the HPLC system 1260 Infinity II (Agilent technology, Santa Clara, CA, USA) with a quaternary pump, a PDA detector, and a vialsampler was used. Polyphenols were separated using a Poroshell 120 EC C-18 column, 4.6 × 100 mm, 2.7 μm and a Poroshell 120 EC-C18 4.6 mm guard-column. The mobile phases were 0.1% H_3_PO_4_ (mobile phase A) and 100% methanol (mobile phase B). The flow was adjusted to 0.8 ml min^−1^. The gradient was used as follows: 0 mL 5% B, 5 min 25% B, 14 min 34% B, 25 min 37% B, 30 min 40% B, 34 min 49% B, 35 min 50% B, 58 min 51% B, 60 min 55% B, 62 min 80% B, 65 min 80% B, 67 min 5% B, 72 min 5% B. A volume of 10 μL of standards and samples was injected into the system. Calibration curves were constructed by analyzing authentic polyphenol standards in different concentration ranges. All standards showed linear calibration curves with *r*^2^ from 0.9927 to 1.0000. Limits of detection (mg L^−1^) and quantification (mg L^−1^) were as follows: (procyanidin B1 (0.8, 2.4), procyanidin B2 (0.6, 1.8), (+)-catechin (0.4, 1.1), (−)-epicatechin (1.3, 4.0), phloretin-2-glucoside (0.2, 0.6), phloretin (0.3, 1.0), chlorogenic acid (0.01, 0.04), *p*-coumaric acid (1.1, 3.5), quercetin-3-glucoside (0.7, 2.2), quercetin-3-galactoside (0.02, 0.07), quercetin-3-rhamnoside (0.8, 2.6), quercetin (0.6, 1.7), cyanidin-3-galactoside (0.2, 0.7). Polyphenols were tentatively identified by comparing the retention times and spectrum of peaks (200 and 600 nm) in extracts with those of authentic standards. Additional confirmation of the identifications was made by spiking polyphenol extracts with authentic standards. Some polyphenols were tentatively identified as phloretin-2-xylosylglucoside, phloretin derivative, chlorogenic acid isomer, *p*-coumaric acid derivative, and quercetin derivative 1 and 2. Those compounds were quantified using calibrations curves of phloretin, chlorogenic acid, *p*-coumaric acid, and quercetin, respectively.

### 2.8. Statistical Analysis

The number of samples for internal and external parameter determination depended on the number of apples of each variety in the quality determination group. For polyphenol determination, the number of samples for each variety was 2 and each was injected into the HPLC system once (*n* = 2). All results were expressed as mean ± standard deviation. For the statistical analysis of the results, the Minitab statistical program (Minitab LLC, State College, PA, USA) and MS Excel (Microsoft, Redmond, WA, USA) were used. Results of internal and external quality traits were shown as histograms to visualize grouping of the data. Correlation was checked by Pearson correlation coefficients. Results of polyphenol amounts were analyzed by using post-hoc Tukey tests to see possible differences within varieties. In addition, principal component analysis (PCA) and dendograms were used to visualize clustering of varieties according to all internal and external quality parameters and polyphenol amounts.

## 3. Results

### 3.1. External and Internal Fruit Quality Parameters

External quality parameters are shown in Table 1. The weights (26–325 g), heights (34–79 mm), widths (41 and 89 mm), fruit shape index values (0.7 and 1.2) of old varieties were similar to those of the commercial variety ‘Idared’ (fruit weight 173 g, height 60 mm, width 75 mm, fruit shape index 0.8). They also agree with literature data [23,38]. External quality parameters are also shown in histograms (Figure S1) where distribution of quality parameters can be seen. ‘Adamova zvijezda’ and ‘Wild apple’ can be highlighted by having the smallest weight, height, and width amongst all apple studied. The majority of other varieties could be in the same group with the commercial variety ‘Idared’ (weight 75 to 175 g (19 varieties), height 49 to 64 mm (17 varieties), width 50 to 80 mm (21 varieties) and shape index 0.67 to 0.87 (22 varieties). Size can play an important role in purchasing apples. Namely, consumer preference for apples depends on many factors, but usually the ideal size accepted is 7.4 to 7.6 cm (according to a study of the Canadian market) [13,39]. Most of the old varieties studied here are of that similar size that consumers prefer, with width 50 to 80 mm (21 varieties) and height 49 to 64 mm (17 varieties). Nine varieties are of specific width between 70 and 80 mm (Figure S1) which would correspond more to the 74 to 76 mm suggested in the study of Hampson et al. (2002) [39]. Fruit shape is also important for consumers to recognize apple varieties [13]. It is determined by the height/width ratio. Only some varieties have fruit shape (ratio height/width) between 0.87 and 1.2 and those varieties are more narrow in their shape and elongated. The majority of old varieties are more wide and flat, with a fruit shape value of 0.67 to 0.87 (Figure S1).

Internal quality parameters are shown in Table 1. Those parameters were similar in old varieties (firmness 3.4 to 13 kg cm^−2^, starch decomposition index (SDI) 1 to 5, soluble solid concentration (SSC) 12–17 °Brix, maturity index (MI) 0.1 to 0.4, total acids 0.3%–1.1%, SSC/TA 13 to 66, pH 2.4–3.8) and in the commercial variety (firmness 5 kg cm^−2^, starch decomposition index 3, soluble solids 12 °Brix, maturity index 0.2, total acids 0.3%, SSC/TA ratio 45, pH 3.4). Quality parameters reported in the literature [19,23,38] correspond to the results of this study. Figure S2 shows the distribution of internal quality parameters. The majority of old varieties were in one group that had values of firmness 3.4–8.2 kg cm^−2^ (22 varieties), starch decomposition index 2.3–5 (24 varieties), maturity index 0.04–0.24 (24 varieties), SSC 13–17.4 °Brix (21 varieties), TA 0.27%–0.68 % (20 varieties), and SSC/TA ratio 13–39 (23 varieties). Only some varieties were outside of those values. In addition, two varieties can be mentioned because they showed lower starch decomposition index and higher maturity index (‘Božićnica 2’ and ‘Zelenika’).

Internal parameters are important for fruit maturity and when deciding about harvest time, and they are also important for consumer acceptance of apples. Fruit firmness is a textural property which decreases with maturity as apples become softer [13]. Apples in this study were mature with regular values of firmness. Starch accumulates in the fruit during growth and starts to degrade with fruit maturity while the fruit is still on the tree [13]. Apples in this study were of various values of starch degradation (1 to 5, most varieties 2.3–5 (24 varieties)) which are in the range of values mentioned in the literature [13]. Soluble solid concentration expresses the amount of sugars, organic acids, and inorganic salts, it increases after storage due to starch degradation, and it is connected to sweetness of apples [13]. On the other hand, total acids express the total amount of organic acids in the fruit. These acids also influence taste and decrease during storage [13]. Old apple varieties in this study showed diversity in those parameters (SSC 12–17.4 °Brix, TA 0.3%–1.1% and SSC/TA ratio 13–66) which can be good for consumer acceptance. Namely, some consumers like sweet apples, some like apples with higher acidity. Furthermore, all apple varieties had between 5–11 seeds, and amongst them 2–10 were healthy and 0.3 to 6.2 were bad (Table 1). Seed weigh was between 0.07 and 0.53 g, and the weight of one seed was between 0.03 and 0.08 g (Table 1).

All external and internal quality parameters and seed characteristics were correlated to see if there is some connection amongst them (Table S1). A positive correlation was found between some basic external fruit characteristics; fruit weight and height (*r*^2^ = 0.77), weight and width (*r*^2^ = 0.94), and height and width (*r*^2^ = 0.72). Those are logical correlations which show that as fruit width and height are increasing, the weight is increasing too. Fruit width is increasing with the increase of fruit height. Fruit height correlates with the shape (*r*^2^ = 0.63), which is also a logical correlations showing the connection of fruit dimensions and fruit shape. Some correlations could also be seen between internal quality parameters. There is a negative correlation between starch decomposition index and maturity index (*r*^2^ = −0.82). As a fruit becomes mature, the starch is decreasing. And there is a negative correlation between TA and SSC/TA (*r*^2^ = −0.77). This is also a logical correlation showing the increase of the SSC/TA ratio as TA is decreasing. Looking at the seeds of apple varieties, some correlations are showing connections between total seeds and the numbers of healthy and bad seeds. Namely, as apples have higher total number of seeds, the number of healthy seeds is increasing too (*r*^2^ = 0.66). As the total healthy seed number is higher, the bad seed number is decreasing (*r*^2^ = −0,82) and the total seed weight is higher (*r*^2^ = 0.78). As the number of bad seeds is decreasing, the total weight of seeds increases (*r*^2^ = −0.65).

**Figure 1:**
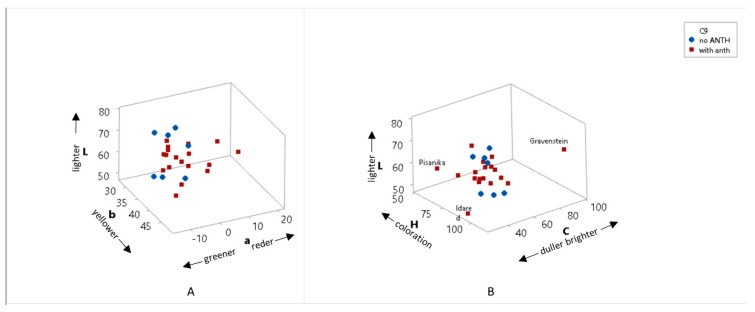
The color of apples (**A**) in CIE L*a*b* coordinates (where L* represents lightness, a* refers to red/green coordinate, b* is yellow/green coordinate), (**B**) in CIE L*C*H* coordinates (where L* represents lightness, C* refers to chroma, H* is the hue angle).

Figure 1 represents the color of apples in the CIE L*a*b* coordinate system (Figure 1A), and in the CIE L*C*H* coordinate system (Figure 1B). Apples are grouped as apples with anthocyanins and without anthocyanins in the peel (Figure 1). In the L*a*b* coordinate system, a* coordinate represents red or green color (+ = redder, − =greener), b* coordinate represents yellow or blue color (+ = yellower, − = bluer), while L* refers to difference in lightness or darkness (+ = lighter, − = darker) on a scale from 0 to 100. The colors of apples are red, green, and yellow and usually apples have a mixture of red and yellow color or green and yellow color (Figure 1A). Apples with anthocyanins in the peel often have a mixture of red and yellow color but also red, yellow, and green color. Varieties without anthocyanins in the peel are of green/yellowish color (Figure 1A). In the L*C*H* system, L* also refers to difference in lightness or darkness (+ = lighter, − = darker) on the scale 0–100, C* represents the difference in chroma (+ = brighter, − =duller), and H* is difference in hue (higher H, lower coloration) (Figure 1B). According to C*, one apple variety stands out having brighter color than others—Gravenstein—while others have similar C* (Figure 1B). In some varieties, the coloration is stronger according to the value of H* (Figure 1B).

In general, the majority of old varieties in this study are of the size that consumers accept. They represent a diversity of shape or taste which can also be positive for consumer acceptance. According to external and internal quality parameters, it can be suggested that old varieties have the quality necessary for acceptance in the market. The majority of old varieties were similar in internal and external quality parameters to the commercial variety ‘Idared’. The color of apples is also very diverse, which also contributes to the acceptance in the market.

### 3.2. Polyphenols in Flesh and Peel

**Figure 2:**
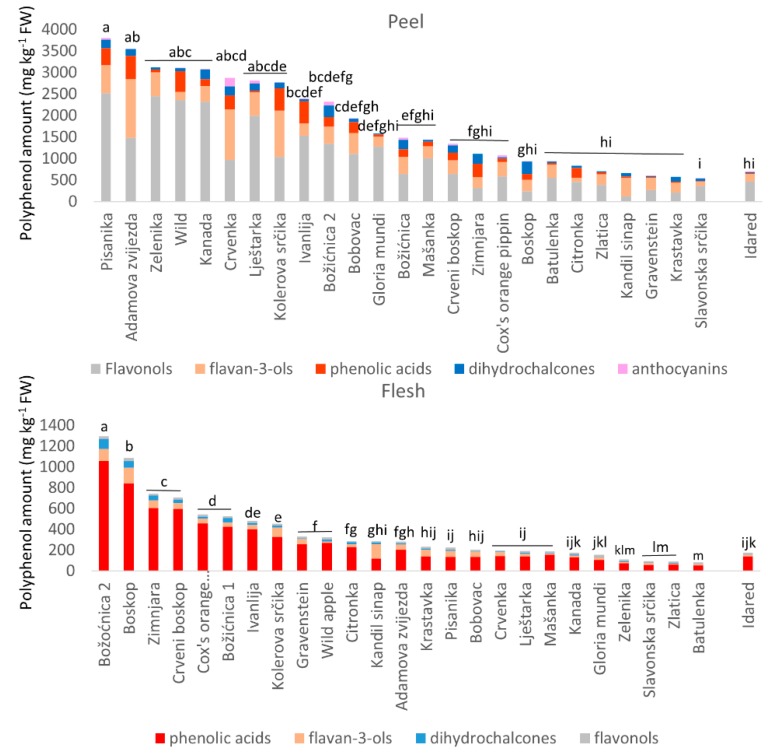
The amount of subgroup of polyphenols (mg kg^−1^ FW) in the peel and flesh of apples. Only the amount of total polyphenols was analyzed with post-hoc Tukey test to see possible differences between varieties (a–m refers to the results of the conducted Tukey test).

Table 2 shows the amounts of individual polyphenols tentatively identified in the peel. These belong to five different polyphenol subgroups: flavan-3-ols (procyanidin B1 and B2, (+)-catechin and (-)-epicatechin), dihydrochalcones (phloretin-2’-glucoside, phloretin-2’-xyloglucoside, and phloretin derivative), phenolic aids (chlorogenic acid and chlorogenic acid isomer), flavonols (quercetin-3-galactoside, quercetin-3-glucoside, quercetin-3-xyloside, quercetin-3-rhamnoside, and quercetin derivatives), and anthocyanins (cyandin-3-galactoside). All these polyphenols have already been reported in the literature [12,23,32]. The amounts of flavan-3-ols (99–1358 mg kg^−1^ FW), dihydrochalcones (19–287 mg kg^−1^ FW), phenolic acids (14–547 mg kg^−1^ FW), flavonols (119–2513 mg kg^−1^ FW), and anthocyanins (0–200 mg kg^−1^ FW) are in agreement with amounts found in the literature [7,38] and with our previous studies [24,25]. Apple varieties that showed the highest total amounts of polyphenols in the peel are ‘Pisanika’, ‘Adamova zvijezda’, ‘Zelenika’, ‘Wild apple’, and ‘Kanada’. It should be mentioned that dihydrochalcones are a specific group of polyphenols found mostly in apples. In fact, apples are their main dietary source [40,41]. Almost all traditional varieties had higher dihydrochalcone content than the commercial variety. Exceptions were ‘Gravenstein’ and ‘Zlatica’. Furthermore, Figure 2 presents the total amount of polyphenol subgroups in the peel of the traditional varieties and the commercial variety. It can be seen that a major contribution to the total amount of polyphenols in the peel comes from flavonols. The majority of traditional apple varieties had higher amounts of polyphenols in the peel than the commercial variety. Varieties do not differ significantly according to the total polyphenol amount in the peel.

Table 3 shows polyphenols tentatively identified in the flesh. Four main subgroups of these polyphenols were found: flavan-3-ols (procyanidin B1 and B2, (+)-catechin and (-)-epicatechin), dihydrochalcones (phloretin-2’-glucoside, phloretin-2’-xylogucoside), phenolic acids (chlorogenic acid, chlorogenic acid isomer, *p*-coumaroylquinic acid, and *p*-coumaric acid derivative), and flavonols (quercetin-3-galactoside, quercetin-3-xyloside, quercetin-3-rhamnoside). These identifications agree with the literature [12,23,32]. The amounts of flavan-3-ols (9–152 mg kg^−1^ FW), dihydrochalcones (2–98 mg kg^−1^ FW), phenolic acids (55–1058 mg kg^−1^ FW), flavonols (8–27 mg kg^−1^ FW) are in agreement with literature data [23,32,38] and with our previous studies [24,25]. Apples that can be highlighted with higher polyphenol amount in the flesh are ‘Božićnica 2’, ‘Boskop’, ‘Zimnjara’, and ‘Crveni boskop’. The majority of traditional varieties had dihydrochalcone content higher than the commercial variety.

Furthermore, Figure 2 presents the total amount of polyphenols from each subgroup in the apple flesh. It is visible that the polyphenol subgroup presenting the highest amount in apple flesh is the phenolic acid subgroup. The majority of traditional apple varieties had higher amounts of polyphenols in the flesh than the commercial variety. Some differences between varieties can be seen according to their total polyphenol amount in the flesh (Figure 2).

As stated, the majority of old, traditional varieties contain higher polyphenol amounts in the flesh and in the peel than the commercial variety. This is a quality trait that should be highlighted for those old varieties. Almost all old, traditional varieties contain higher dihydrochalcone content.

Although the amounts in the peel of old, traditional varieties do not differ statistically, some varieties can be highlighted according to higher polyphenol content in the peel, namely, ‘Pisanika’, ‘Adamova zvijezda’, ‘Zelenika’, ‘Wild apple’, and ‘Kanada’. Moreover, the flesh of the varieties ‘Božićnica 2’, ‘Boskop’, ‘Zimnjara’, and ‘Crveni boskop’ contained high content of polyphenols.

### 3.3. Differences in Varieties

**Figure 3:**
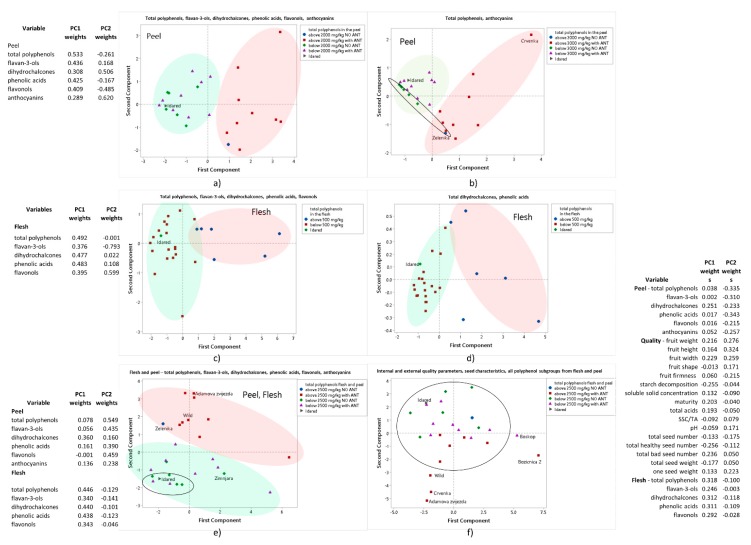
Principal component analysis of (**a**) the amounts of all polyphenol subgroups in the peel, (**b**) the amount of total polyphenols and total anthocyanins in the peel, (**c**) the amount of all polyphenol subgroups in the flesh, (**d**) the amount of total phenolic acids and total dihydrochalcones in the flesh, (**e**) the amount of total polyphenols of all subgroups in the flesh and peel, (**f**) all external and internal quality traits, all seed characteristics, the amount of all polyphenol subgroups in the flesh and peel. NO ANT—apples with no anthocyanins in the peel, WITH ANT—apples with anthocyanins in the peel. Values for principal component 1 and 2 were also shown.

To visualize some differences between old varieties according to their internal and external quality characteristics and polyphenol content, principal component analysis (PCA) was used to analyze the data (Figure 3).

The PCA analysis was first used to analyze the total amounts of all polyphenol subgroups in the peel of all varieties (Figure 3a) and to analyze total polyphenols and total anthocyanins in the peel (Figure 3b). Total anthocyanins were chosen for Figure 3b because they are a subgroup that separates varieties into varieties with red/redish color or no red color. Varieties clustered into two clusters according to the amount of total polyphenols in the peel. Namely, varieties with more than 2000 mg kg^−1^ FW of total polyphenols in the peel are grouped in one, and all other in a second cluster. Green apples can be grouped into one cluster that overlaps with two other clusters (Figure 3b).

The total amounts of polyphenol subgroups in the flesh were also analyzed with PCA (Figure 3c). In addition, the amounts of two of the most important subgroups in the flesh, namely, total phenolic acids and total dihydrochalcones, were also analyzed (Figure 3d). According to these PCA analyses of the flesh, again two clusters can be seen. One for varieties with total polyphenol amount in the flesh above 500 mg kg^−1^ FW, and the other with lower amounts.

An interesting feature of the PCA analyses using all the polyphenol subgroups (Figure 3a for peel and Figure 3c for flesh) is the nature of the first principle component. The first principle component defines the single composite feature (as a weighted combination of the amounts of all polyphenol subgroups) that best explains the variability in the data. Separately, in the peel case (Figure 3a) and in the flesh case (Figure 3c), the PCA finds the first principle component to give positive weights to all the subgroups consistent with the observed importance of total polyphenols as a distinguishing characteristic of the apples. In contrast, the second (and subsequent) principle components find weights of combination of both positive and negative signs, that is, these are formed by differences in amounts of polyphenol subgroups.

Finally, all polyphenol subgroups from the peel and flesh were analyzed with one PCA (Figure 3e). Varieties again grouped into two clusters. In general, one cluster consisted of varieties with the amount of total polyphenols in the peel and flesh above 2500 mg kg^−1^ FW, and the other one with varieties with lower amount of total polyphenols. Apples with no anthocyanins in the peel clustered close within the group of apples with lower polyphenol content. The exceptions here are ‘Zimnjara’ and Zelenika’. In all PCA diagrams (Figure 3a to 3e), the commercial variety ‘Idared’ was in the cluster within old varieties with lower amounts of polyphenols. This suggests that some traditional, old varieties are similar to a commercial variety, but some do differ. The varieties that do differ and that belong to the cluster with higher amounts of polyphenols are ‘Pisanika’, ‘Kanada’, ‘Crvenka’, ‘Lještarka’, ‘Kolerova srčika’, ‘Ivanlija’, ‘Božićnica 2’, ‘Wild apple’, ‘Adamova zvijezda’, and ‘Zelenika’ (Figure 3e). Wild apples are also here in the cluster with apples of higher polyphenol content.

In this joint treatment, in one PCA of all polyphenol subgroups from the peel and flesh (Figure 3e), it is interesting to note that the first two principle components it finds essentially correspond to the total polyphenols in the flesh (according to the weights of the first component) and the total polyphenols in the peel (according to the weights of the second component). An interesting exception is that peel dihydrochalcones also is given significant weight in the first principle component. This PCA reinforces the importance of these polyphenol features (predominately in accordance with the respective flesh and peel totals) in explaining the variability amongst apples of all the polyphenol subgroup data.

Finally, principal component analysis was used to analyze all data, internal and external quality parameters, seed characteristics, amount of polyphenol subgroup in the flesh and peel (Figure 3f). Here, clustering of most of the varieties in one cluster can be seen, with several varieties that differ from others, ‘Božićnica 2’, ‘Boskop’, ‘Wild apple’, ‘Adamova zvijezda’, and ‘Crvenka’. The weights of the principal components show a balance of importance of the internal/external quality characteristics and the polyphenol subgroup amounts in the flesh and peel.

**Figure 4:**
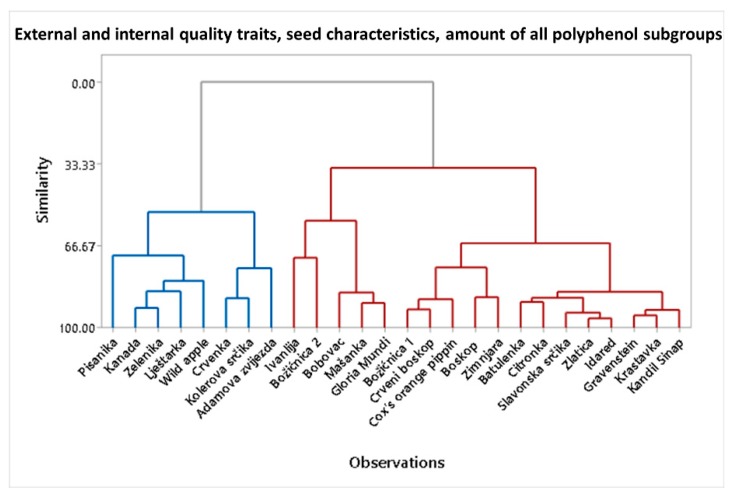
Dendogram of all fruit internal and external quality parameters, all seed parameters, and the amounts of all polyphenol subgroups in flesh and peel. It shows the clustering of apple varieties into two clusters (the group colored blue corresponds to higher polyphenol content and the group colored red corresponds to lower polyphenol content).

All internal and external parameters, seeds characteristics, and amount of polyphenol subgroups were also analyzed with a dendogram representation of clustering. In the dendogram (Figure 4), two large clusters can also be seen. The first cluster contains some varieties that have high polyphenol content in the peel and flesh like ‘Pisanika’, ‘Kanada’, ‘Zelenika’, ‘Lještarka’, ‘Wild apple’, ’Adamova zvijezda’, ‘Crvenka’, and ‘Kolerova srčika’. These results are consistent with the results of the PCA analysis where the same varieties clustered in one cluster (Figure 3a–e). Those mentioned varieties are also somewhat different from the commercial variety. As seen from dendogram, other varieties are in the cluster together with the commercial variety. The dendogram is also interesting at the lowest levels of the diagram (Figure 4), where it reveals which pairs and triplets of varieties are most alike.

In general, it can be suggested that varieties cluster into two main clusters according to total polyphenol content, one is a cluster of varieties with higher total polyphenol content (above 2500 mg kg^−1^ FW) and the other with varieties with a lower total polyphenol content. These results were consistent with principal component analysis and also with dendograms. But the final analysis with principal component analysis, which took into account all internal and external quality parameters and polyphenol content, showed a difference of the group of varieties ‘Božićnica 2’, ‘Boskop’, ‘Wild apple’, ‘Adamova zvijezda’, and ‘Crvenka’ when compared to other varieties. The other varieties clustered into a second group.

## 4. Discussion

Old, traditional varieties of apples have shown many positive nutritional characteristics in earlier studies. For instance, it was found that traditional apples in Portugal have more fiber, proteins, sugar, β-carotene, vitamin E, Mg, and polyphenolic compounds in comparison to some exotic commercial varieties (such as ‘Fuji’, ‘Gala Galaxy’, ‘Golden’, ‘Reineta Parda,’ and ‘Starking’) [27]. Lanzerstorfer et al. [42] studied mineral content, phosphate and trace elements, and polyphenol content in apple juices made from mostly old, traditional apple varieties. Great variations were found between varieties according to the content of these elements. And it was concluded that selected apple varieties can serve for apple products with a focus on health-beneficial effects [42]. Positive characteristics of old, traditional apple cultivars are also their sensory characteristics (firmness, juiciness, taste, shape, color, and odor). Sensory characteristics of traditional varieties were well accepted by consumers and by a sensory panel in an earlier study [27]. Traditional, old varieties in this study showed richness in their quality traits which can be important for consumer acceptance. The majority of varieties in this study are of the size that consumers accept. They represent a diversity of shape, sugar and acid content, color, appropriate firmness. According to external and internal quality parameters, it can be suggested that old, traditional varieties have the quality necessary for acceptance in the market.

Studies are beginning to show the beneficial effects of old varieties and their polyphenols. Serra et al. [43] demonstrated anti-proliferative activity of polyphenols from traditional and exotic commercial apple varieties from Portugal against human colon and gastric cancer cells, as well as anti-oxidative activity. In addition, apples can cause allergic reaction [44], but it was found that old varieties can be better tolerated than new varieties by individuals who developed intolerance to apples [32]. This was connected to a higher amount of polyphenols [32]. It has also been shown that less allergenic genotypes of apples are old varieties [33]. Since in this study, almost all old, traditional varieties had higher polyphenol content than commercial variety, it might be suggested that this is their beneficial quality trait with a potential of positive bioactivities. Earlier studies also suggested that phenolic compounds from apples can be used in dermal formulations due to many beneficial characteristics such as antioxidant or antimicrobial activity [9]. Traditional varieties could be a good source of polyphenols for dermal formulations since they have a higher polyphenol content than commercial varieties.

Dihydrochalcones are a specific group of polyphenolic compounds from apples with potential to reduce blood glucose levels [45,46] which is helpful in diabetes prevention. Oral antidiabetic drugs based on phloridzin conjugate were suggested as well [47]. Old apples in this study have shown much higher dihydrochalcone content (Table 2 and Table 3) than a commercial variety. This is also one of important reasons to consider these varieties for more extensive growth.

Earlier studies have shown that there are differences in old apple varieties [34]. But studies of genetic diversity revealed that there are also many synonymous amongst old apple varieties. For example, 43.3% of apple varieties were synonymous in the collection in Italy [34]. In the Dutch collections, that was 32% [48]. It was also highlighted that synonyms have the same genetic origin, but they can still show differences in some characteristics like peel color which also deserves the attention. The reason for many synonyms is the spreading of varieties through travelling or migrations of people through centuries. Apples studied here are harvested in Croatia, but probably surrounding countries have the same apple varieties with a different name. Nonetheless, apples in this study deserve attention due to the high number of those varieties, and their diversity in internal and external quality characteristics. The significance in preserving traditional varieties can also be seen in their importance for enhancing nutraceutical composition of other apple varieties by innovative breeding strategies or for new functional foods development [49] or, as mentioned earlier, for abiotic and biotic stress resistance which can be important for apple growers [50].

Due to all of that, old, traditional varieties of apples are very valuable fruits. Apples studied here for the first time, showed some differences according to quality characteristics and polyphenols in the flesh and peel. Those differences should be further studied with genetic tests of the varieties to separate those unique and especially valuable for preservation.

## 5. Conclusions

In twenty-five traditional, indigenous apple varieties, sixteen different quality traits and color were determined for the first time and compared to a commercial variety ‘Idared’, the commercial variety most grown in Croatia [51]. This is also the continuation of our earlier studies [24,25]. It has been shown that apples have external (weight, height, width, shape) and internal quality parameters (firmness, starch degradation, soluble solid concentration, total acids) that can be suitable for more extensive growth and their quality traits might be attractive to consumers. The majority of varieties contain more polyphenols in the peel in comparison to the commercial variety. The flesh of some varieties contains more polyphenols than the commercial variety. Furthermore, dihydrochalcones which are specific apple polyphenols, connected with beneficial effects on the human health, have been found in much higher content in old, traditional varieties than in the commercial variety. Those quality traits (amounts of total polyphenols and dihydrochalcones) should be highlighted for the traditional varieties. There are some differences in the traditional varieties that can be ascribed to higher amounts of polyphenols, and the varieties with such higher amounts can be highlighted as ‘Pisanika’, ‘Kanada’, ‘Crvenka’, ‘Lještarka’, ‘Kolerova srčika’, ‘Ivanlija’, ‘Božićnica 2’, ‘Wild apple’, ‘Adamova zvijezda’, and ‘Zelenika’. The differences between varieties should be further studied with genetic tests to separate those unique and especially valuable for preservation. The results of the present paper should be incentive for the reevaluation of traditional, neglected varieties and their importance in the market, especially according to quality traits important to consumers. The results should help encourage the preservation of these varieties. According to the content of polyphenols, it may be suggested that the old varieties might be used for foods with added value.

## Figures and Tables

**Table 1:** Basic external and internal fruit quality parameters and seed characteristics.

Variety	External Quality Traits				Internal Quality Traits							Seeds				
													Number		Weight	
	Weightt	Height	Width	Shape	Firmness	SDI	SSC	MI	TA	SSC/TA	pH	Total	Healthy	Bad	Total	1 Seed
	g	mm	mm	index	kg/cm^2^		°Brix		%						g	g
Crvenka	77 (12)	48 (3)	58 (3)	0.8 (0.0)	9 (1)	5 (0)	15 (1)	0.1 (0.0)	0.4 (0.0)	39 (3)	2.4 (0.1)	10.33 (1.75)	7.33 (1.03)	3.00 (1.90)	0.31 (0.03)	0.04 (0.01)
Crveni boskop	167 (18)	62 (3)	73 (3)	0.9 (0.1)	7 (1)	4 (0)	16 (1)	0.1 (0.0)	1.1 (0.1)	15 (1)	3.3 (0.1)	7.50 (2.17)	3.40 (1.58)	4.10 (2.08)	0.19 (0.08)	0.06 (0.01)
Pisanika	94 (15)	49 (5)	65 (3)	0.8 (0.0)	4 (1)	5 (0)	16 (1)	0.1 (0.0)	0.5 (0.1)	39 (12)	3.6 (0.2)	7.20 (1.30)	6.00 (0.71)	1.20 (1.10)	0.23 (0.04)	0.04 (0.00)
Lještarka	96 (11)	50 (4)	59 (2)	0.9 (0.1)	7 (1)	3 (1)	15 (1)	0.1 (0.0)	0.4 (0.1)	38 (7)	3.5 (0.1)	7.80 (1.30)	3.20 (1.30)	4.60 (2.07)	0.13 (0.05)	0.04 (0.01)
Božićnica 1	152 (7)	52 (2)	78 (2)	0.7 (0.0)	6 (1)	4 (1)	14 (1)	0.1 (0.0)	0.6 (0.1)	22 (3)	3.2 (0.1)	6.83 (1.47)	4.67 (1.37)	2.17 (2.14)	0.22 (0.07)	0.05 (0.00)
Božićnica 2	188 (36)	58 (4)	81 (6)	0.7 (0.0)	7 (0)	1 (0)	14 (1)	0.4 (0.1)	0.7 (0.1)	19 (2)	3.3 (0.0)	7.40 (2.07)	3.30 (1.89)	4.10 (1.29)	0.17 (0.09)	0.05 (0.01)
Cox’ Orange Pippin	102 (18)	50 (5)	64 (3)	0.8 (0.1)	9 (1)	4 (1)	12 (1)	0.2 (0.0)	0.9 (0.1)	13 (2)	3.1 (0.1)	8.00 (0.67)	5.20 (1.69)	2.80 (1.48)	0.43 (0.12)	0.08 (0.00)
Ivanlija	111 (24)	63 (7)	63 (5)	1.0 (0.1)	6 (1)	3 (0)	16 (1)	0.1 (0.0)	0.3 (0.0)	59 (4)	3.8 (0.1)	9.00 (0.82)	8.75 (0.96)	0.25 (0.50)	0.42 (0.09)	0.05 (0.01)
Boskop	189 (13)	61 (3)	76 (2)	0.8 (0.0)	8 (1)	4 (0)	17 (1)	0.1 (0.0)	0.5 (0.1)	39 (2)	3.2 (0.0)	8.00 (2.16)	2.67 (1.50)	5.33 (1.89)	0.10 (0.09)	0.04 (0.01)
Bobovac	139 (26)	61 (5)	70 (4)	0.9 (0.0)	6 (0)	5 (0)	16 (0)	0.1 (0.0)	0.8 (0.1)	21 (4)	3.3 (0.1)	7.00 (1.62)	3.75 (1.43)	3.25 (0.82)	0.22 (0.06)	0.06 (0.00)
Slavonska srčika	126 (21)	55 (4)	69 (4)	0.8 (0.0)	7 (1)	5 (0)	15 (0)	0.1 (0.0)	0.5 (0.0)	31 (1)	3.6 (0.0)	4.80 (1.30)	1.60 (0.89)	3.20 (0.84)	0.07 (0.03)	0.05 (0.00)
Kolerova srčika	211 (16)	66 (2)	80 (2)	0.8 (0.0)	7 (1)	4 (0)	16 (1)	0.1 (0.0)	1.0 (0.1)	17 (2)	3.2 (0.0)	8.60 (1.67)	2.40 (0.55)	6.20 (1.64)	0.14 (0.02)	0.06 (0.01)
Batulenka	161 (8)	60 (2)	70 (3)	0.9 (0.0)	4 (1)	5 (0)	14 (1)	0.1 (0.0)	0.5 (0.1)	26 (9)	3.3 (0.0)	6.20 (0.76)	5.50 (1.04)	0.70 (0.71)	0.25 (0.04)	0.05 (0.00)
Gravenstein	95 (16)	50 (4)	63 (4)	0.8 (0.0)	7 (7)	4 (4)	12 (12)	0.1 (0.1)	0.5 (0.0)	23 (2)	3.3 (3.3)	8.67 (8.67)	6.22 (6.22)	2.44 (2.44)	0.28 (0.28)	0.04 (0.04)
Mašanka	81 (15)	48 (3)	58 (3)	0.8 (0.0)	9 (1)	4 (1)	16 (1)	0.2 (0.0)	0.5 (0.1)	31 (6)	3.3 (0.1)	9.00 (1.00)	7.60 (1.14)	1.40 (1.14)	0.43 (0.08)	0.06 (0.01)
Kanada	171 (26)	59 (4)	76 (3)	0.8 (0.0)	7 (1)	3 (1)	15 (1)	0.2 (0.1)	0.6 (0.1)	25 (4)	3.3 (0.3)	8.30 (1.64)	4.60 (1.71)	3.70 (1.57)	0.27 (0.09)	0.06 (0.01)
Kandil Sinap	143 (18)	79 (4)	64 (3)	1.2 (0.0)	5 (1)	5 (0)	13 (1)	0.1 (0.0)	0.4 (0.0)	34 (4)	3.3 (0.0)	5.73 (2.02)	5.73 (2.02)	2.60 (1.12)	0.34 (0.12)	0.06 (0.01)
Citronka	78 (16)	43 (2)	60 (4)	0.7 (0.0)	7 (2)	5 (0)	13 (1)	0.1 (0.0)	0.6 (0.1)	21 (3)	3.3 (0.1)	10.00 (0.00)	8.60 (0.55)	1.40 (0.55)	0.42 (0.07)	0.05 (0.01)
Zimnjara	149 (13)	72 (23)	72 (5)	1.0 (0.4)	5 (0)	3 (1)	16 (1)	0.1 (0.0)	0.9 (0.1)	17 (2)	3.1 (0.1)	7.40 (2.07)	7.00 (1.58)	0.40 (0.89)	0.34 (0.07)	0.05 (0.00)
Zlatica	159 (34)	57 (5)	74 (6)	0.8 (0.0)	3 (1)	5 (0)	12 (0)	0.1 (0.0)	0.5 (0.0)	25 (2)	3.5 (0.1)	10.75 (2.99)	10.25 (3.30)	0.50 (1.00)	0.34 (0.11)	0.03 (0.01)
Gloria Mundi	325 (40)	75 (4)	89 (5)	0.9 (0.1)	7 (1)	3 (1)	14 (2)	0.2 (0.1)	0.4 (0.1)	37 (13)	3.4 (0.1)	8.80 (0.84)	3.40 1(.34)	5.40 (1.34)	0.26 (0.12)	0.07 (0.01)
Zelenika	229 (64)	60 (6)	83 (6)	0.7 (0.0)	8 (1)	2 (1)	14 (1)	0.4 (0.1)	0.6 (0.1)	23 (2)	3.2 (0.1)	6.10 (1.29)	3.20 (1.03)	2.90 (1.66)	0.26 (0.08)	0.08 (0.01)
Krastavka	110 (24)	50 (5)	63 (4)	0.8 (0.1)	3 (0)	5 (0)	16 (2)	0.0 (0.0)	0.4 (0.2)	66 (69)	3.5 (0.0)	7.90 (1.73)	3.10 (1.60)	4.80 (2.53)	0.12 (0.07)	0.04 (0.02)
Adamova zvijezda	46 (63)	34 (9)	50 (11)	0.7 (0.0)	13 (2)	5 (0)	13 (1)	0.2 (0.0)	0.5 (0.1)	27 (8)	3.4 (0.1)	9.00 (3.61)	8.75 (1.15)	0.25 (2.52)	0.27 (0.03)	0.03 (0.02)
Wild apple	26 (4)	34 (3)	41 (2)	0.8 (0.0)	4 (1)	4 (0)	15 (0)	0.1 (0.0)	0.6 (0.0)	13 (0)	3.3 (0.0)	9.40 (2.61)	6.60 (2.61)	2.80 (1.48)	0.19 (0.08)	0.03 (0.01)
Idared	173 (21)	60 (3)	75 (3)	0.8 (0.0)	5 (1)	3 (0)	12 (0)	0.2 (0.0)	0.3 (0.1)	45 (9)	3.4 (0.0)	9.80 (1.23)	8.70 (1.70)	1.10 (0.99)	0.53 (0.99)	0.06 (0.00)

SDI—starch decomposition index; SSC—soluble solid concentration; MI—maturity index; TA—total acids as % of malic acid, numbers in brackets are standard deviations.

**Table 2:** The amount of polyphenols in the peel of old apple varieties (mg kg^−1^ fresh weight).

	Flavan-3-ols				Total	Dihydrochalcones				Phenolic Acids		Total	Flavonols						Total	Anth	Total
	PCB1	C	PCB2	EC		phder	ph2xglclu	ph2glc	Total	chac	chac is		q3gal	q3glc	qder1	qder2	q3xyl	q3rh		cy3gal	
Crvenka	88 (42)	436 (5)	181 (1)	474 (2)	1179	25 (0)	61(0)	126 (0)	212	30 (16)	289 (2)	319	507 (2)	218 (0)	58 (0)	13 (0)	129 (0)	39 (0)	964	200 (1)	2874 (71)
Crveni boskop	88 (16)	113 (1)	53 (1)	62 (1)	316		33(0)	135 (1)	168	116 (1)	62 (1)	178	470 (6)	21 (3)	29 (0)	10 (0)	65 (0)	49 (0)	644	41 (0)	1347 (31)
Pisanika		418 (2)	93(1)	142 (1)	653	35 (1)	52(1)	108 (2)	195	284 (3)	112 (1)	396	1403 (28)	492 (6)	100 (2)	25 (0)	216 (4)	277 (5)	2513	44 (1)	3801 (58)
Lještarka		245 (2)	91 (3)	206 (7)	542	18 (3)		151 (1)	169	42 (33)		42	1244 (11)	120 (2)	139 (1)	19 (0)	294 (3)	178 (2)	1994	64 (1)	2811 (69)
Božićnica 1	37 (9)	161 (1)	59 (0)	143 (56)	400	29 (0)		193 (0)	222	55 (0)	121 (0)	176	354 (0)	125 (0)	35 (0)	9 (0)	72 (0)	44 (0)	639	42 (0)	1479 (66)
Božićnica 2	32 (0)	146 (0)	54 (0)	161 (0)	393	35 (0)	37 (0)	195 (1)	267	139 (0)	85 (0)	224	732 (1)	280 (0)	79 (0)	14 (0)	156 (1)	81 (0)	1342	93 (1)	2319 (4)
Coxs orange	32 (1)	152 (17)	50 (11)	98 (17)	332	2 (0)	5 (0)	25 (3)	32	39 (5)	44 (6)	83	257 (35)	79 (6)	33 (2)	31 (7)	75 (5)	108 (7)	583	46 (9)	1076 (131)
Ivanlija		103 (27)	21 (2)	163 (0)	287	5 (0)	3 (0)	39 (0)	47	293 (1)	224 (1)	517	801 (2)	409 (0)	54 (0)	10 (0)	103 (0)	155 (0)	1532	17 (0)	2400 (33)
Boskop		101 (13)	62 (3)	105 (2)	268	33 (1)		254 (2)	287	73 (1)	65 (2)	138	135 (0)	16 (1)	17 (0)	5 (0)	35 (0)	32 (0)	240	12 (0)	945 (25)
Bobovac	24 (2)	250 (11)	85 (1)	125 (9)	484	6 (1)	17 (5)	57 (13)	80	122 (34)	138 (40)	260	515 (14)	110 (7)	71 (5)	25 (4)	194 (24)	189 (6)	1104	12 (6)	1940 (180)
Slavonska srčika	15 (1)	32 (11)	15 (3)	40 (13)	102	4 (2)	7 (4)	42 (7)	53	14 (2)	4 (2)	18	159 (6)	100 (14)	16 (2)	11 (0)	41 (4)	32 (3)	359	4 (1)	536 (75)
Kolerova srčika		462 (2)	175 (0)	440 (0)	1077	12 (1)	12 (2)	109 (1)	133	300 (1)	219 (1)	519	471 (3)	106 (3)	92 (1)	10 (0)	207 (3)	152 (5)	1038	8 (0)	2775 (23)
Batulenka	31 (8)	133 (19)	62 (7)	79 (20)	305	5 (0)		23 (3)	28	42 (1)		42	365 (18)	59 (4)	25 (1)	8 (0)	48 (2)	47 (2)	552	3 (1)	930 (86)
Gravenstein	16 (7)	123 (30)	73 (8)	75 (20)	287	1 (0)	5 (1)	14 (4)	20	15 (8)	8 (1)	23	116 (8)	24 (0)	19 (0)	6 (0)	55 (1)	46 (0)	266	9 (1)	605 (89)
Mašanka		122 (5)	48 (0)	110 (26)	280	5 (1)	5 (3)	35 (7)	45	73 (23)	32 (6)	105	565 (202)	180 (28)	48 (5)	12 (2)	98 (8)	103 (15)	1006	5 (0)	1441 (331)
Kanada	58 (11)	139 (51)	66 (20)	101 (63)	364	37 (9)	15 (5)	179 (61)	231	161 (47)		161	1321 (64)	516 (22)	89 (11)	19 (1)	160 (18)	211 (24)	2316	3 (0)	3075 (407)
Kandil Sinap	51 (7)	100 (116)	52 (10)	235 (34)	438	8 (0)	5 (1)	66 (5)	79	27 (1)		27	38 (2)	23 (0)	9 (0)	5 (0)	13 (1)	31 (1)	119		663 (178)
Citronka	34 (1)	26 (0)	14 (0)	25 (0)	99			51 (0)	51	208 (6)	13 (1)	221	220 (1)	138 (0)	22 (0)	5 (0)	40 (0)	33 (0)	458		829 (9)
Zimnjara	37 (0)	88(13)	39(2)	92(4)	256		55 (1)	177 (3)	232	196 (1)	112 (1)	308	85 (1)	79 (1)	34 (5)	5 (1)	78 (1)	31 (0)	312		1108 (34)
Zlatica		139(19)	51(2)	52(2)	242	2(1)	4 (0)	13 (0)	19	23 (0)	28 (0)	51	178 (1)	64 (0)	27 (0)	8 (0)	68 (0)	43 (1)	388		700 (26)
Gloria Mundi	23 (0)	116 (27)	39 (20)	53 (11)	231	4 (0)		22(3)	26	51(38)		51	648 (210)	313 (76)	47 (8)	11 (2)	110 (16)	147 (19)	1276		1584 (430)
Zelenika	25 (3)	232 (3)	93 (3)	200 (21)	550	9 (1)	1 (0)	44 (2)	54	68 (4)		68	1411 (32)	349 (5)	135 (2)	24 (1)	281 (9)	251 (9)	2451		3123 (95)
Krastavka	32 (1)	97 (33)	41 (8)	56 (33)	226	18 (1)	18 (4)	67 (11)	103		35 (7)	35	63 (23)	53 (12)	20 (4)	6 (1)	48 (11)	19 (4)	209		573 (153)
Adamova zvijezdajezda	102 (11)	519 (6)	170 (2)	567 (22)	1358	8 (1)	36 (1)	107 (8)	151	259 (15)	288 (17)	547	681 (354)	534 (239)	50 (8)	19 (5)	130 (30)	72 (7)	1486	5 (1)	3547 (727)
Wild		134 (26)	59 (15)		193	10 (6)	6 (0)	51 (28)	67	349 (155)	130 (4)	479	693 (359)	1156 (656)	101 (45)	16 (6)	204 (80)	185 (73)	2355	17 (19)	3111 (1472)
Idared	26 (11)	85 (10)	29 (3)	44 (10)	184	2 (0)		22 (3)	24	14 (14)		14	238 (50)	26 (4)	34 (7)	10 (1)	84 (19)	70 (13)	462	15 (1)	699 (146)

Polyphenols tentatively identified as: PCB1—procyanidin B1, C—(+)-catechin, PCB2—procyanidin B2, EC—(-)-epicatechin, phder—phloretin derivative, ph2xglc—phloretin-2’-xyloglucoside, ph2glc—phloretin-2’-glucoside, chac—chlorogenic acid, chac is—chlorogenic acid isomer, q3gal—quercetin-3-galactoside, q3glc—quercetin-3-glucoside, q der1—quercetin derivative 1, q der 2—quercetin derivative 2, q3xyl—quercetin-3-xyloside, q3rh—quercetin-3-rhamnoside, cy3gal—cyanidin-3-galactoside, Anth—anthocyanins, numbers in brackets are standard deviations.

**Table 3:** The amount of polyphenols in the flesh of old apple varieties (mg kg^−1^ fresh weight).

	Flavan-3-ols					Dihydrochalcones			Phenolic Acid					Flavonols				
	PCB1	C	PCB2	EC	Total	ph2xglc	ph2glc	Total	chac	chac is	pcq	pcder	Total	q3gal	q3xyl	q3rh	Total	Total
Crvenka	5 (0)	6 (8)	10 (2)	11 (3)	33 (13)	3 (0)	6 (0)	9 (0)	60 (0)	36 (0)	24 (0)	25 (0)	145 (0)			8 (0)	8 (0)	195 (13)
Crveni boskop	2 (0)		14 (0)	40 (1)	56 (1)	8 (0)	22 (0)	30 (0)	472 (5)	62 (1)	24 (0)	40 (0)	598 (6)	7 (0)	4 (0)	9 (0)	20 (0)	704 (7)
Pisanika	10 (6)	26 (1)	11 (0)	9 (0)	56 (7)	5 (0)	8 (0)	13 (0)	89 (1)	22 (0)		23 (0)	134 (1)	6 (0)	4 (0)	8 (0)	18 (0)	221 (8)
Lještarka	2 (0)		8 (0)	13 (1)	23 (1)		10 (0)	10 (0)	79 (0)	19 (0)	20 (0)	23 (0)	141 (0)	4 (0)	4 (0)	9 (0)	17 (0)	191 (1)
Božićnica 1	11 (1)	6 (0)	6 (0)	19 (4)	42 (5)	19 (0)	23 (0)	42 (0)	275 (3)	82 (1)	26 (0)	40 (0)	423 (4)	6 (0)	4 (0)	10 (0)	20 (0)	527 (9)
Božićnica 2	20 (2)	27 (1)	14 (0)	51 (6)	112 (9)	41 (0)	57 (0)	98 (0)	802 (3)	184 (1)	21 (0)	51 (0)	1058 (4)	10 (0)	4 (0)	12 (0)	26 (0)	1294 (13)
Cox orange pippin	11 (1)		6 (0)	30 (0)	47 (1)	5 (0)	13 (5)	18 (5)	331 (7)	54 (2)	29 (0)	43 (0)	457 (9)	5 (0)	5 (0)	10 (0)	20 (0)	542 (15)
Ivanlija			9 (0)	33 (1)	42 (1)	5 (0)	13 (0)	18 (0)	333 (15)	40 (13)		27 (0)	400 (28)	11 (0)	4 (0)	7 (0)	22 (0)	482 (29)
Boskop	27 (0)		17 (0)	108 (0)	152 (0)	24 (0)	44 (0)	68 (0)	715 (4)	65 (0)		60 (0)	840 (4)	10 (0)	5 (0)	12 (0)	27 (0)	1087 (4)
Bobovac			7 (2)	41 (7)	48 (9)	3 (0)	4 (1)	7 (1)	103 (22)	4 (3)		27 (1)	134 (26)	2 (0)	5 (0)	9 (0)	16 (0)	205 (36)
Slavonska srčika	16 (1)		8 (1)		24 (2)		2 (0)	2 (0)	3 (0)	2 (0)	25 (0)	26 (0)	56 (0)	1 (0)	4 (0)	8 (0)	13 (0)	95 (2)
Kolerova srčika	10 (2)	26 (0)	11 (0)	46 (4)	93 (6)	5 (0)	12 (0)	17 (0)	236 (7)	37 (13)	23 (0)	29 (0)	325 (20)	3 (0)	5 (0)	11 (0)	19 (0)	454 (26)
Batulenka	2 (0)		8 (0)		10 (0)	1 (0)	1 (0)	2 (0)	17 (1)	10 (3)		28 (1)	55 (5)		4 (0)	8 (0)	12 (0)	79 (5)
Gravenstein	10 (4)		14 (3)	24 (12)	48 (19)	7 (0)	4 (1)	11 (1)	179 (35)	20 (4)	27 (0)	32 (2)	258 (41)		5 (0)	9 (0)	14 (0)	331 (61)
Kandil Sinap	18 (5)	41 (37)	14 (5)	67 (9)	140 (56)	6 (6)	8 (1)	14 (7)	48 (5)	14 (1)	27 (0)	29 (1)	118 (7)		4 (0)	9 (0)	13 (0)	285 (70)
Citronka	2 (0)		9 (0)	20 (0)	31 (0)	7 (0)	11 (0)	18 (0)	181 (1)	20 (0)		26 (0)	226 (1)	3 (0)		8 (0)	11 (0)	286 (1)
Zimnjara	12 (0)	11 (1)	12 (0)	40 (0)	75 (1)	30 (0)	17 (0)	47 (0)	453 (2)	107 (1)		43(0)	603 (3)	5 (0)	4 (0)	7 (0)	16 (0)	741 (4)
Zlatica			9 (0)		9 (0)	2 (0)	3 (0)	5 (0)	37 (0)			27 (0)	64 (0)		4 (0)	9 (0)	13 (0)	91 (0)
Mašanka	9 (1)				9 (1)	2 (0)	5 (0)	7 (0)	106 (2)	19 (4)		30 (0)	155 (6)	3 (0)	5 (0)	9 (0)	17 (0)	188 (7)
Kanada	7 (2)		10 (1)		17 (3)	3 (1)	9 (1)	12 (2)	50 (13)	23 (7)	25 (0)	30 (2)	128 (22)	4 (3)	4 (0)	10 (0)	18 (3)	175 (30)
Gloria Mundi	5 (1)		9 (3)	9 (0)	23 (4)		4 (0)	4 (0)	39 (1)	13 (0)	25 (0)	30 (0)	107 (1)	5 (0)	5 (0)	11 (0)	20 (0)	154 (5)
Zelenika	7 (2)		8 (2)		15 (4)	2 (1)	4 (0)	6 (1)	44 (9)	2 (1)		26 (1)	72 (11)	2 (1)	4 (1)	10 (0)	16 (2)	109 (18)
Krastavka	10 (2)	18 (1)	17 (2)	21 (3)	66 (8)	6 (0)	6 (5)	12 (5)	52 (10)	24 (15)	28 (1)	33 (1)	137 (27)	4 (0)	4 (0)	10 (0)	18 (0)	233 (40)
Adamova zvijezda	9 (3)	10 (1)	7 (0)	22 (1)	48 (5)	2 (1)	5 (0)	7 (1)	117 (14)	33 (4)	26 (0)	29 (0)	205 (18)	2 (0)	5 (0)	9 (0)	16 (0)	276 (24)
Wild			11 (0)		11 (0)	6 (0)	15 (1)	21 (1)	183 (9)	25 (0)	29 (0)	32 (0)	269 (9)	5 (0)	5 (0)	9 (0)	19 (0)	320 (10)
Idared	4 (0)		6 (0)	9 (0)	19 (0)	1 (0)	3 (0)	4 (0)	68 (3)	13 (0)	27 (0)	29 (0)	137 (3)	2 (0)	4 (1)	9 (0)	15 (1)	175 (4)

Polyphenols tentatively identified as: PCB1—procyanidin B1, C—(+)-catechin, PCB2—procyanidin B2, EC—(-)-epicatechin, ph2xglc—phloretin-2’-xyloglucoside, ph2glc—phloretin-2’-glucoside, chac—chlorogenic acid, chac is—chlorogenic acid isomer, pcq—*p*-coumaroylquinic acid, pcder—*p*-coumaric acid derivative; q3gal—quercetin-3-galactoside, q3xyl—quercetin-3-xyloside, q3rh—quercetin-3-rhamnoside, number in brackets are standard deviations.

## Supplementary Materials

The following are available online at https://www.mdpi.com/2304-8158/9/1/52/s1, Figure S1: Histogram of basic external fruit quality characteristics with marked commercial apple variety (‘Idared’), Figure S2: Histogram of basic internal fruit quality characteristics with marked commercial apple variety (‘Idared’), Table S1: Pearson correlation coefficient for internal and external fruit characteristics and seed characteristics.
