# Supplementary material for: Traditional, Indigenous Apple Varieties, a Fruit with Potential for Beneficial Effects: Their Quality Traits and Bioactive Polyphenol Contents
Source: Foods. 2020 Jan 5;9(1):52. doi: 10.3390/foods9010052 (PMC7022233; doi:10.3390/foods9010052)
Supplement: Supplementary file 1 [file foods-09-00052-s001.pdf]

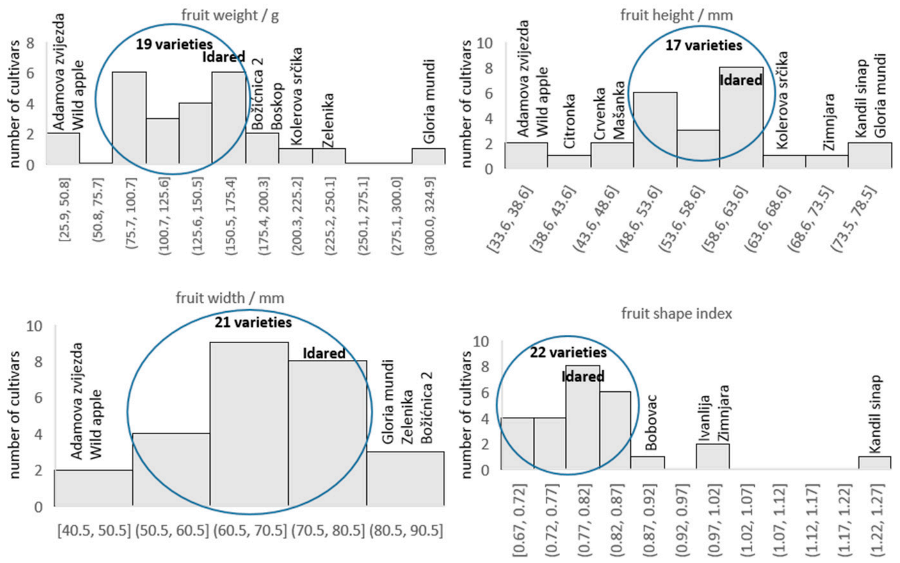

Figure S1. Histogram of basic external fruit quality characteristics with marked commercial apple variety (Idared)

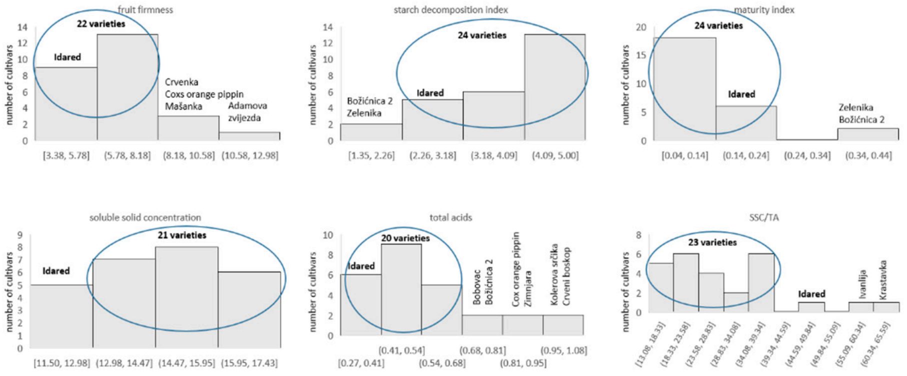

Figure S2. Histogram of basic internal fruit quality characteristics with marked commercial apple variety (Idared).

Table S1. Pearson correlation coefficient for internal and external fruit characteristics and seed characteristics

		Fruit characteristics					Fruit quality						Seeds				
													Number			weight	
		weight g	height mm	width mm	shape index	firmness (kg cm^{-2})	SDI	SSC (°Brix)	MI	TA (%)	SSC/TA	pH	total	healthy	bad	total g	1 seed g
external	weight (g)	1.00															
	height (mm)	**0.77**	1.00														
	width (mm)	**0.94**	**0.72**	1.00													
	shape index	0.05	**0.63**	-0.08	1.00												
internal	firmness (kg cm^{-2})	-0.09	-0.27	-0.11	-0.30	1.00											
	SDI	-0.55	-0.35	-0.54	0.07	-0.19	1.00										
	SSC (°Brix)	0.04	0.14	0.00	0.21	-0.13	-0.07	1.00									
	MI	0.39	0.08	0.40	-0.32	0.51	**-0.82**	-0.24	1.00								
	TA (%)	0.14	0.11	0.22	-0.10	0.17	-0.16	0.06	0.18	1.00							
	SSC/TA	-0.03	0.09	-0.07	0.20	-0.23	0.08	0.31	-0.24	**-0.77**	1.00						
	pH	0.04	0.07	0.03	0.06	-0.39	0.03	0.02	-0.17	-0.25	0.33	1.00					
seeds	total number	-0.22	-0.35	-0.27	-0.22	0.14	0.06	-0.17	-0.08	-0.18	0.09	-0.22	1.00				
	number of healthy	-0.46	-0.30	-0.44	0.06	0.00	0.27	-0.38	-0.21	-0.30	0.07	-0.03	**0.66**	1.00			
	number of bad	0.45	0.24	0.35	-0.05	0.07	-0.26	0.34	0.17	0.21	0.00	-0.15	-0.16	**-0.82**	1.00		
	total weight(g)	-0.14	0.03	-0.12	0.18	0.10	-0.04	-0.47	0.06	-0.16	0.04	-0.12	0.47	**0.78**	**-0.65**	1.00	
	1 seed weight(g)	0.58	0.50	0.55	0.10	0.21	-0.51	-0.13	0.52	0.33	-0.19	-0.10	-0.30	-0.35	0.27	0.27	1.00

SDI – starch decomposition index; SSC – soluble solid concentration, MI – maturity index; TA – total acids (% of malic acid)
